# Abdominal cerebrospinal fluid pseudocyst occurring 21 years after ventriculoperitoneal shunt placement: a case report

**DOI:** 10.1186/1471-2482-13-27

**Published:** 2013-07-08

**Authors:** Atsumi Tamura, Dai Shida, Kyosuke Tsutsumi

**Affiliations:** 1 Department of Surgery, Tokyo Metropolitan Bokutoh Hospital, 4-23-15 Koto-bashi, Sumida-ku, Tokyo 1308575, Japan; 2 Colorectal Surgery Division, National Cancer Center Hospital, 5-1-1 Tsukiji, Cyuo-ku, Tokyo 1040045, Japan; 3 Department of Neurosurgery, Tokyo Metropolitan Bokutoh Hospital, 4-23-15 Koto-bashi, Sumida-ku, Tokyo 1308575, Japan

**Keywords:** Abdominal pseudocyst, Ventriculoperitoneal shunt

## Abstract

**Background:**

Ventriculoperitoneal shunt (VPS) placement is an established procedure for the treatment of hydrocephalus of diverse etiologies in children and adults. Abdominal cerebrospinal fluid pseudocyst, which is potentially life threatening, is a rare complication and usually occurs during childhood. However, with increasing longevity following successful treatment, it can also occur in adults.

**Case presentation:**

Here we describe a 22-year-old man who was admitted to our hospital because of diffuse abdominal distention. A VPS was placed 21 years earlier to treat hydrocephalus secondary to spina bifida. Abdominal computed tomography (CT) revealed a homogeneous low-density fluid collection adjacent to the VPS catheter tip, causing stomach obstruction. Thus a peritoneal pseudocyst around VPS was suspected and emergency laparotomy was performed. The large mass was localized in the left upper abdomen between the stomach and mesentery of the transverse colon, exactly at the omental bursa. The cystic mass was opened and 1500 ml of clear fluid was drained; the distal end of the VPS was repositioned outside the mass. Thus, an abdominal cerebrospinal fluid pseudocyst as a complication of VPS was diagnosed.

**Conclusion:**

Gastroenterological surgeons should be aware of this possible complication, and this complication should be considered during differential diagnosis of an acute abdomen complaint.

## Background

Ventriculoperitoneal shunt (VPS) placement is a surgical procedure performed to relieve high intracranial pressure caused by hydrocephalus of diverse etiologies in children and adults [1]. Various extracranial complications of VPS may be seen, such as tube disconnection, infection, omental clogging, abdominal visceral perforation, and bowel obstruction [1-3]. Abdominal cerebrospinal fluid pseudocyst is a rare but important complication of VPS, with its incidence ranging from less than 0.33% to 6.8% [1-6]. This complication is characterized by the collection of cerebrospinal fluid in the peritoneal cavity containing the distal end of the VPS catheter and is surrounded by a wall composed of fibrous tissue without an epithelial lining. This complication is extremely rare in adults [1,2,4], with most cases reported in children [3,6-8]. Here we present a case of abdominal cerebrospinal fluid pseudocyst that occurred 21 years after VPS placement and appears to be the longest interval reported until date in the English literature.

## Case presentation

A 22-year-old man presented to our hospital with progressive abdominal distention and nausea. A VPS had been placed 21 years earlier to treat hydrocephalus secondary to spina bifida. Shunt revision was performed only once when he was 10 months old, and no other abdominal surgery was performed after this. He had no history of malignancy or pancreatic or liver disease. Physical examination revealed a large elastic palpable mass in the upper abdomen. No neurological change was observed. His white blood cell count and amylase serum levels were normal. The patient underwent supine abdominal radiography and unenhanced abdominal computed tomography (CT).

**Figure 1:**
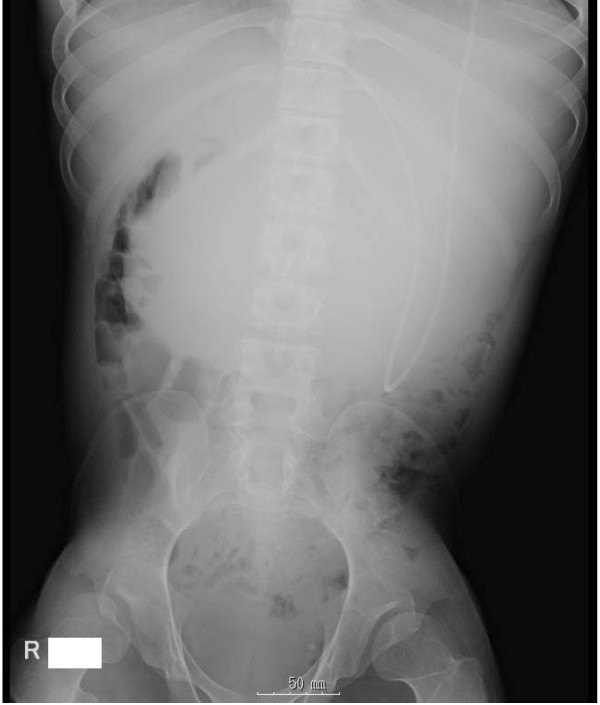
**Abdominal radiography showing the VPS catheter and a soft**-**tissue mass located in the upper abdomen.**

**Figure 2:**
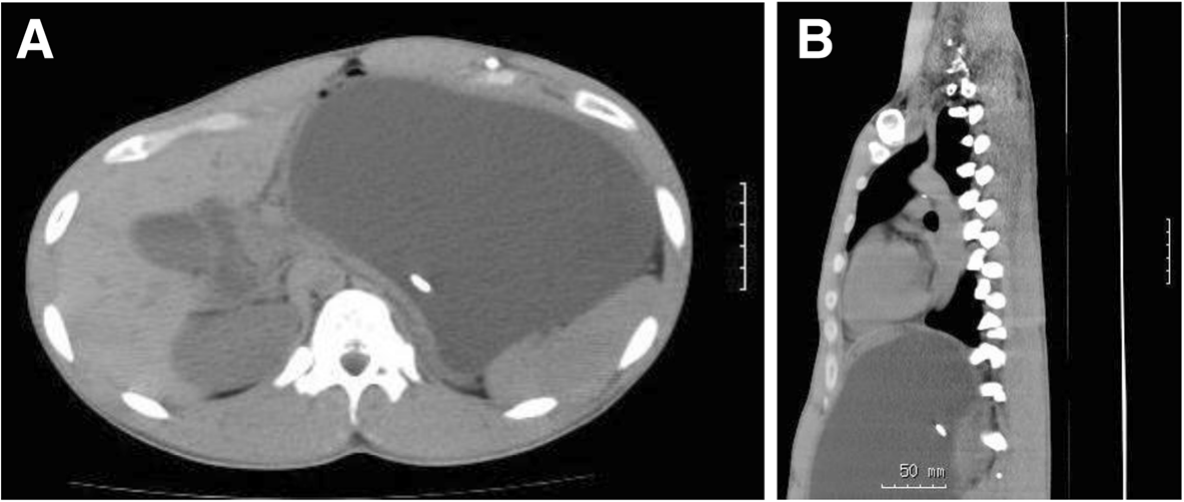
**Abdoinal CT (A: axial, B: sagital) showing the fluid collection adjacent to the VPS catheter tip. A,B** Abdominal CT showing the fluid collection adjacent to the VPS catheter tip.

Supine abdominal radiography revealed the VPS in the left upper quadrant and a soft-tissue mass in the upper abdomen (Figure 1). Abdominal CT revealed homogeneous low-density fluid collection. The VPS catheter tip was adjacent to the fluid collection, suggesting a giant cystic lesion around the shunt catheter (Figure 2A,B). The cystic mass was independent of the pancreas.

**Figure 3:**
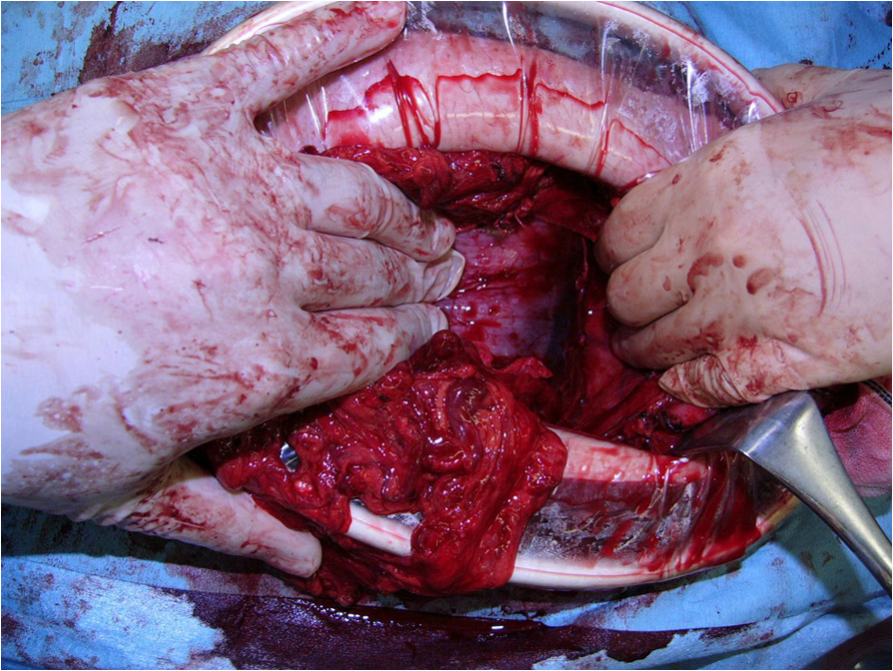
Intraoperative finding presenting a large mass located at the omental bursa.

**Figure 4:**
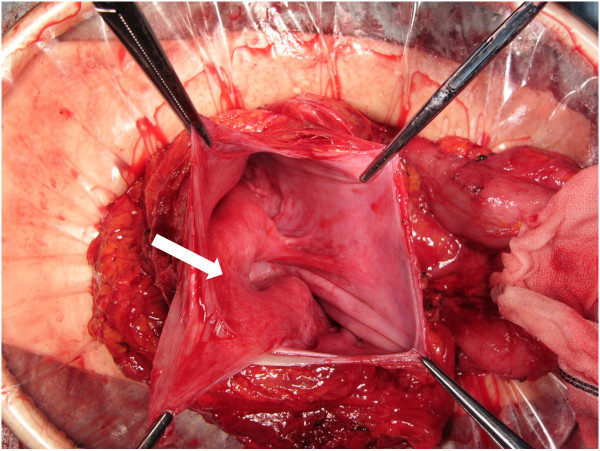
**Intraoperative finding presenting the inner cavity of the pseudocyst.** Wall of the pseudocyst consisted of the posterior wall of stomach (arrow) and colonic mesentery.

Thus, a peritoneal pseudocyst around the VPS causing stomach obstruction was suspected and emergency laparotomy was performed. The large mass was localized in the left upper abdomen between the stomach and transverse colon mesentery, exactly at the omental bursa (Figure 3). After the diffuse abdominal adhesions were removed, the cystic mass was opened and 1500 ml of clear fluid was drained. A part of the pseudocyst wall, consisting of the posterior wall of the stomach and colonic mesentery, was resected completely to open the pseudocyst (Figure 4). The distal end of the VPS was placed inside the mass. Thus, pseudocyst as a complication of VPS was diagnosed. After examination of the cerebrospinal fluid from the tip of VPS catheter, we replaced the VPS catheter to another position in the abdomen. Histopathological examination of the resected specimen revealed fibrous tissue with inflammatory cell infiltration. After surgery, his appetite recovered and was discharged on the 8th postoperative day in a good condition. The postoperative course has been uneventful for 4 months after his last surgery.

The findings of fluid analysis were similar to those of cerebrospinal fluid analysis, with negative cultures and absence of microbial flora.

### Discussion

VPS is the most common treatment procedure for hydrocephalus; however, it is not free of complication. Abdominal cerebrospinal fluid pseudocyst is a rare, but potentially life-threatening complication of VPS placement. It is seen as a thin-walled cystic mass around the shunt tip, which is filled with cerebrospinal fluid. The wall is composed of fibrous tissue without epithelial lining. The underlying mechanisms involved in the formation of the cerebrospinal fluid pseudocyst remain unknown; however, inflammation, either sterile or infectious, is usually regarded as the main causative factor [1]. In our case, there was diffuse abdominal adhesion, suggesting a history of previous abdominal infection.

The symptoms of abdominal cerebrospinal fluid pseudocysts in adult patients are abdominal pain, distention, and a palpable abdominal mass. Thus, adults predominantly present with abdominal signs only. On the other hand, in pediatric patients, symptoms of shunt malfunction, such as headache, nausea, and vomiting are more common [9].

CT indicates a definitive diagnosis of abdominal cerebrospinal fluid pseudocyst, revealing a large fluid-filled collection delimited by a thin wall adjacent to the catheter tip. Differential diagnoses of an abdominal cystic mass include abdominal abscess, lymphocele, seroma, cystic lymphangioma, cystic mesothelioma, mesenteric cyst, benign cystic teratoma, cystic spindle-cell tumor, pancreatic pseudocyst, and duplication cyst [2]. In our case, the absence of infectious symptoms and lack of any inflammatory changes in surrounding tissues makes the diagnosis of the abscess highly unlikely. Furthermore, the absence of a history of pancreatic disease makes diagnosis of pancreatic pseudocyst unlikely. The preoperative diagnosis of pseudocyst around the VPS was made considering the clinical setting and the relationship of the cyst with VPS.

The time between the last VPS surgery and collection of abdominal cerebrospinal fluid has been reported to range from 3 weeks to 10 years [4]. Recently, both Pernas *et al*. [2] and Sena *et al*. [1] reported a case of abdominal pseudocyst formation 15 years after VPS placement, both of which seem to be the longest period reported previously in the English literature. In our case, the last VPS surgery was performed 21 years before the beginning of current symptoms, and no other abdominal surgery was performed during that period. This appears to be the longest period reported until date in the English literature.

Treatment of abdominal cerebrospinal fluid seems controversial. Therefore, many treatment algorithms have been described, such as laparotomy with wide excision of cystic walls, paracentesis and aspiration of the cystic fluid, CT-guided or ultrasound-guided aspiration of the pseudocyst. In the present case, exploratory laparotomy and excision of the cystic walls with fluid drainage was performed. Recently, laparosopic management, which is a useful tool, has been reported [10]; however, in our case, it seemed impossible to use this tool because of diffuse abdominal adhesion.

## Conclusion

In conclusion, this report described an adult case of a huge abdominal pseudocyst as a complication of VPS. Not only neurosurgeons or pediatricians but also physicians should be aware of this possible complication because early diagnosis and treatment would improve the clinical outcome and reduce the patient’s suffering and distress.

### Consent

Written informed consent was obtained from the patient for publication of this Case report and any accompanying images. A copy of the written consent is available for review by the Series Editor of this journal.

## Abbreviations

VPS: Ventriculoperitoneal shunt; CT: Computed tomography.

## Competing interests

The authors declare that they have no competing interests. No financial support has been received.

## Authors’ contributions

TA and TK collected the data, performed the treatment and wrote the paper; SD was responsible for writing the paper and its supervision. All authors read and approved the final manuscript.

## Pre-publication history

The pre-publication history for this paper can be accessed here:

http://www.biomedcentral.com/1471-2482/13/27/prepub
